# Decentralization and immunization program in a single-party state: the case of the Lao People’s Democratic Republic

**DOI:** 10.1186/s41182-024-00601-8

**Published:** 2024-05-07

**Authors:** Phonevilay Viphonephom, Sengchanh Kounnavong, Daniel Reinharz

**Affiliations:** 1 Department of Social and Preventive Medicine, Laval University, Quebec City, QC Canada; 2 Lao Tropical and Public Health Institute (Lao TPHI), Ministry of Health, Vientiane, Lao PDR

**Keywords:** Decentralization, Immunization program, Neo-institutional theory, Single-party state, Lower-middle income country, Lao PDR

## Abstract

**Background:**

The Lao People’s Democratic Republic (Lao PDR), a lower-middle-income country, lags behind other Southeast Asian countries in immunization coverage for children under two years of age. The organization of health services is a key determinant of the functionality of immunization programs. However, this aspect, and in particular its decentralization component of the healthcare system, has never been studied.

**Methods:**

A case study in the Lao National Immunization Program was performed using a neo-institutional theory-based conceptual framework, highlighting the structure (rules, laws, resources, etc.) and interpretative schemes (dominant beliefs and ideas) that underlie the state of decentralization of the healthcare system that support the conduct of the immunization program. Twenty-two semi-structured interviews were conducted with representative actors from various government levels, external donors, and civil society, in four provinces. Data were complemented with information retrieved from relevant documents.

**Results:**

The Lao healthcare system has a deconcentrated form of decentralization. It has a largely centralized structure, albeit with certain measures promoting the decentralization of its immunization programs. The structure underlying the state of centralization of immunization services provided is coherent with a shared dominant interpretive scheme. However, the rapid economic, technical, and educational changes affecting the country suggest that the coherence between structure and interpretative schemes is bound to change.

**Conclusion:**

Unprecedented opportunities to access quality higher education and the use of social networks are factors in Lao PDR that could affect the distribution of responsibilities of the different levels of government for public health programs such as the National Immunization Program.

**Supplementary Information:**

The online version contains supplementary material available at 10.1186/s41182-024-00601-8.

## Introduction

Decentralization involves the transfer of authority and responsibility for public functions from a central government to subnational or lower levels of government [1]. It is a national, political, legislative, institutional and fiscal process that involves power-sharing arrangements and decision-making within different levels of government [1, 2]. From a healthcare perspective, decentralization can be beneficial in particular for countries with a geographically diverse population. It can improve the efficiency, equity, accessibility and quality of healthcare service delivery [3, 4]. Granting local governments more control over healthcare resources and decision-making through decentralization can lead to better tailored services that are more effective in meeting the specific needs of the local population.

Indeed, the state of decentralization is recognized as a key political and institutional factor influencing immunization programs, especially in developing countries. The literature highlights the fact that giving immunization responsibilities to local governments enhances accountability for service delivery and boosts immunization coverage rates, notably in low-resource settings with diverse population demographics [5, 6].

Lao PDR is a lower-middle-income country situated in Southeast Asia. It has a population of seven million spread over a territory equivalent to the United Kingdom. It has been, since 1975, a single-party socialist state lead by the Lao People’s Revolutionary Party (LPRP) that is the sole legal political party in the country. As such, Lao PDR is led by the Marxist-Leninist concept of democratic centralism aimed at achieving one of the main goals of socialist parties, the social equality across the country. This ideological stance partially explains why the healthcare system in Lao PDR is considered to be of the deconcentration type, a type in which certain responsibilities are delegated to local governments while the ultimate control remains centralized. In a deconcentration system, local branches of central institutions are field offices or executing directives without autonomous decision-making authority [7, 8]. Several studies and reviews on the decentralized state of Lao PDR were conducted, primarily in the early 2000s [7–27]. While many focused on public administration and fiscal decentralization [7–24], a few also examined the decentralized healthcare system and its financial and administrative situation [25–27]. These studies highlighted challenges, especially in remote regions, related to the distribution of human and financial resources, which affect the capacity and effectiveness of healthcare system at the local levels (provincial, district and health center levels) in developing their own public health programs.

In a country like Lao PDR which contains, on a geographical basis, significant ethnic diversity, devolving power to provincial and district governments could, theoretically at least, more effectively address the behavioral factors of the of the population and health professionals, which pose obstacles to the optimization of vaccination coverage. Beliefs about the origin of diseases and the ability of vaccines to prevent them, and the negative experiences suffered by individuals from ethnic minorities above all, during their contact with public health services, have indeed been described as factors associated with low vaccination coverage [28–31]. Yet, the Lao immunization program is a national priority since the establishment of the Lao PDR. The program aims to achieve a 95% immunization rate among one-year-old children by 2025 [29]. No recent data are available on the current rate, but a 2022 study reported that merely 68% of children aged between 12 and 35 months were fully immunized [32]. This study shows that although significant efforts have been made to improve child vaccination indicators in the country, Lao PDR still lags behind other Southeast Asian countries. Diphtheria, tetanus, pertussis (DTP3) immunization coverage for children under 2 years of age for example was 79% in Lao PDR in 2020. This is lower than the coverage rates in Myanmar (84%), Cambodia (92%), and Vietnam (94%) [33]. Immunization coverage rates in Lao PDR reveal significant disparities among different population groups. According to the latest national survey conducted in 2017, full immunization coverage among young people aged 12–23 varies considerably across provinces, ranging from 9% in Xaysomboune Province to 75% in Borikhamxay Province [34]. Moreover, substantial disparities exist based on economic status, with DPT coverage rates ranging from 81% in the wealthiest quintile to 42% in the poorest quintile [35].

Key factors influencing immunization coverage span both demand and supply sides. On the demand side, challenges include geographical barriers, cultural and linguistic beliefs among ethnic minorities, poverty, and low literacy levels, which limit awareness and acceptance of immunization benefits among caregivers. On the supply side, shortages of skilled healthcare workers in remote areas, particularly in delivering outreach services to hard-to-reach populations [29, 32, 36]. Additionally, deficiencies in healthcare workers’ interpersonal communication skills, often stemming from language barriers and inadequate training in engaging with ethnic communities, exacerbate these challenges [29]. Furthermore, reported issues with vaccine provision and maintenance during transportation and storage at health facilities compound these challenges. These factors finally affect not only immunization coverage, but also seroconversion rates among vaccinated children's [36–38]. Other factors less explored in the literature on the Lao PDR immunization program include the decentralization of immunization services and programs. While there is growing recognition of its potential importance for improving immunization programs and its functioning [28, 35], this aspect remains relatively understudied in Lao PDR.

## Method

### Conceptual framework

The conceptual framework used in this study is based on Neo-institutional Theory (NIT). This theory allows interpreting how institutions’ programs are formed, implemented and functioning under the influence of institutionalized factors in their environment. NIT brings an understanding of these programs as the result of social and cultural systems embedded within the institutional context that influence the actors' interpretations of rules and regulations based on their views and beliefs toward organizations [39, 40].

This theory has been employed to examine decentralized healthcare systems in various countries, seeking to comprehend how the decentralized context influences the effectiveness of health programs and systems [41, 42].

NIT has been the source of different approaches that have been developed to address organization issues in healthcare systems. One of the approaches focuses on the dialectic between structure and interpretive schemes. The structure consists of laws, regulations, and formal and informal rules that underlie the functioning of organizations [43, 44]. Interpretive schemes consist of collective and dominant ideas, norms, values, and beliefs originating among and toward actors in the organizational context [43, 45]. The dialectic between structure and interpretive schemes provides insight into the social dynamics of interactions between institutionalized factors. This insight allows interpreting how institutionalized organizations function in a specific societal context and legitimize the structure that underlies their offer of services to the population.

### Study design

An ethnographic case study design was employed to investigate the social and organizational determinants that underlie the formal–informal structure, as well as the dominant beliefs, values, and behaviors of groups of actors involved in the immunization program in Lao PDR [46, 47]. Data were collected through document reviews and interviews with actors engaged in the immunization program across various administrative levels in the study sites, assessing the perceptions of actors toward the functioning of the program at the national level.

### Data collection

#### Study sites

Data were collected in the capital and in three provinces, Luangnamtha in the North, Xayabury in the Center and Saravan in the South. In these sites, the prevalence of complete vaccination according to the national vaccination schedule is 38%, 68%, 49% and 61%, respectively, compared to a national average of 48% [34]. One notes that the capital serves as the headquarters for the Ministry of Health (MoH), the Maternal and Child Health Center (MCHC), and all reference institutions related to maternal and child health. It also serves as the base for the headquarters of external donors, non-governmental organizations (NGOs), and civil society organizations (CSOs). The recruitment period for this study was between 15 January and 30 June 2022.

#### Interview guide

An interview guide was developed based on the study’s conceptual framework. It allowed discussing the following themes: immunization coverage rates, power distribution among government levels in Lao PDR, and the roles of various actors in immunization functioning across different authorized levels. The discussion explored formal and informal structures, including policies and regulations, and delved into informants' beliefs and perceptions influencing power dynamics in the immunization program (Additional file 1).

#### Sources of information

Two sources of information were applied for this study: semi-structured interview with key actors and document reviews.

#### Informants

Key participants for this research study were selected based on specific criteria: (1) having a formal position in their organization related to the immunization program for at least one year; (2) being authorized by the organization they represent to speak on its behalf; and (3) being able to communicate in one of the languages spoken by the interviewer, Lao (official language), English, or French.

A preliminary list of participants was drawn by one co-author (SK). A purposeful sampling approach was used to ensure the inclusion of all relevant vaccination organizations in the study provinces [48]. At the end of each interview, informants were asked to suggest additional individuals or organizations for further insights into the topic under discussion. A snowball sampling method was subsequently employed to enhance the diversity of information pool [46, 49].

In accordance with Lao Ministry of Health regulations, the sampling process began with consent from relevant organization directors. Designated individuals within these organizations were then contacted for individual interviews, with a consent form detailing the project sent via WhatsApp or email. Informants had the option of face-to-face or online interviews lasting 30–60 min, with consent for audio recording obtained. Handwritten notes were taken during the interviews to capture the discussion flow and nonverbal clues.

#### Documents

The information provided by the informants was supplemented by documents written in English, Lao, or French pertaining to immunization programs and the decentralized healthcare system in Lao PDR. These documents included published scientific papers, official documents, materials provided by informants, and documents retrieved from the websites of the informants’ organizations.

### Data analysis

The analysis employed an inductive–deductive approach [46, 50]. Data collected from interviews and documents underwent analysis using NVivo 11 software. The data analysis process involved four main steps: (1) coding (from phrases, sentences, or paragraphs); (2) categorization (into themes or concepts); (3) coding of themes according to the conceptual framework; and (4) comparison of the emerging information with findings from other scientifically published studies on similar topics.

### Data validation

The validity of the findings was ensured through four elements. Credibility was achieved by triangulating information from multiple sources. Transferability was ensured by providing a detailed description of participants, the research process, and the study context. Reliability was determined by conduction of independent analyses by two researchers (PV and DR) and searching for a consensus between them in case of discrepancies. Confirmability was ensured through a detailed and transparent record of the research process, including notes on data collection, analysis, interpretation, and discussion [46, 51].

## Results

Semi-structured interviews were conducted with 22 individuals representing diverse groups, including government institutions, the Global Alliance for Vaccine and Immunization (GAVI), National Immunization Technical Advisory Groups (NITAGS), Non-Government Organizations (NGOs) and Civil Society Organizations (CSOs). The majority of participants were women (12 out of 22). The average age was 46, with a range of 35 to 61. Among the 22 participants, one worked at the central level (or Ministry of Health) of the healthcare system, six at the provincial level, seven at the district level, and three at health centers. There was one participant from each of the following organizations: GAVI, NITAGs, NGOs, and two CSOs. One interview was conducted in French, the others in Lao language (Table 1).

**Table 1:** Basic characteristics of interviewed participants

Number of participants		22
Female		12
Age	Mean (min–max)	46 (35–61)
Organization	Healthcare institution at central level	1
	Healthcare institution at provincial level	6
	Healthcare institution at district level	7
	Health center	3
	GAVI	1
	NITAG	1
	NGOs	1
	CSOs	2
Languages of interview	Lao	21
	French	1

In addition, eight documents were analyzed: (1) Fiscal Decentralization in the People’s Democratic Republic of Lao; (2) Health System Review; (3) National Immunization Programme: Updated Comprehensive Multi-Year Plan Lao PDR 2019–2023; (4) Toward Sustainable Financing for Immunization Coverage in Lao PDR; (5) National Immunization Programme: Comprehensive multi year plan 2007–2011; (6) Promulgation of the Amended Law on the Government of the Lao People’s Democratic Republic; (7) International Review of the Expanded Programme on Immunization; and (8) Financial Sustainable Plan of National Immunization Program [9, 25, 28–30, 52–54].

### Power sharing of government actors in the health sector

In Lao PDR, the national government, under the supervision of the LPRP Politburo, holds primary authority over all forms of power sharing between government levels regarding the political, administrative, and fiscal systems within the country [54].

Provincial governors are appointed by the President of the country based on the Prime Minister’s recommendation for a five-year term. Officially, responsibilities at the provincial level encompass the management of political, economic, sociocultural affairs, and human resources. In practice, however, the dynamics of power between the national and provincial governments are shaped by a complex interplay of human and political factors that extend beyond constitutional provisions [9, 54]. Above all, the leadership of the LPRP holds significant influence within the political system, extending to all levels of government and to their respective sectors, including healthcare.

Lao PDR's healthcare system operates across three administrative levels: central (Ministry of Health, or MoH), provincial (provincial health departments, or PHDs), and district (district health offices, or DHOs). The MoH coordinates a range of health services and manages information, the workforce, finances, and international collaborations at all levels of governance [25].

### Structure and interpretive schemes influencing the functioning of immunization program in Lao PDR

**Fig. 1:**
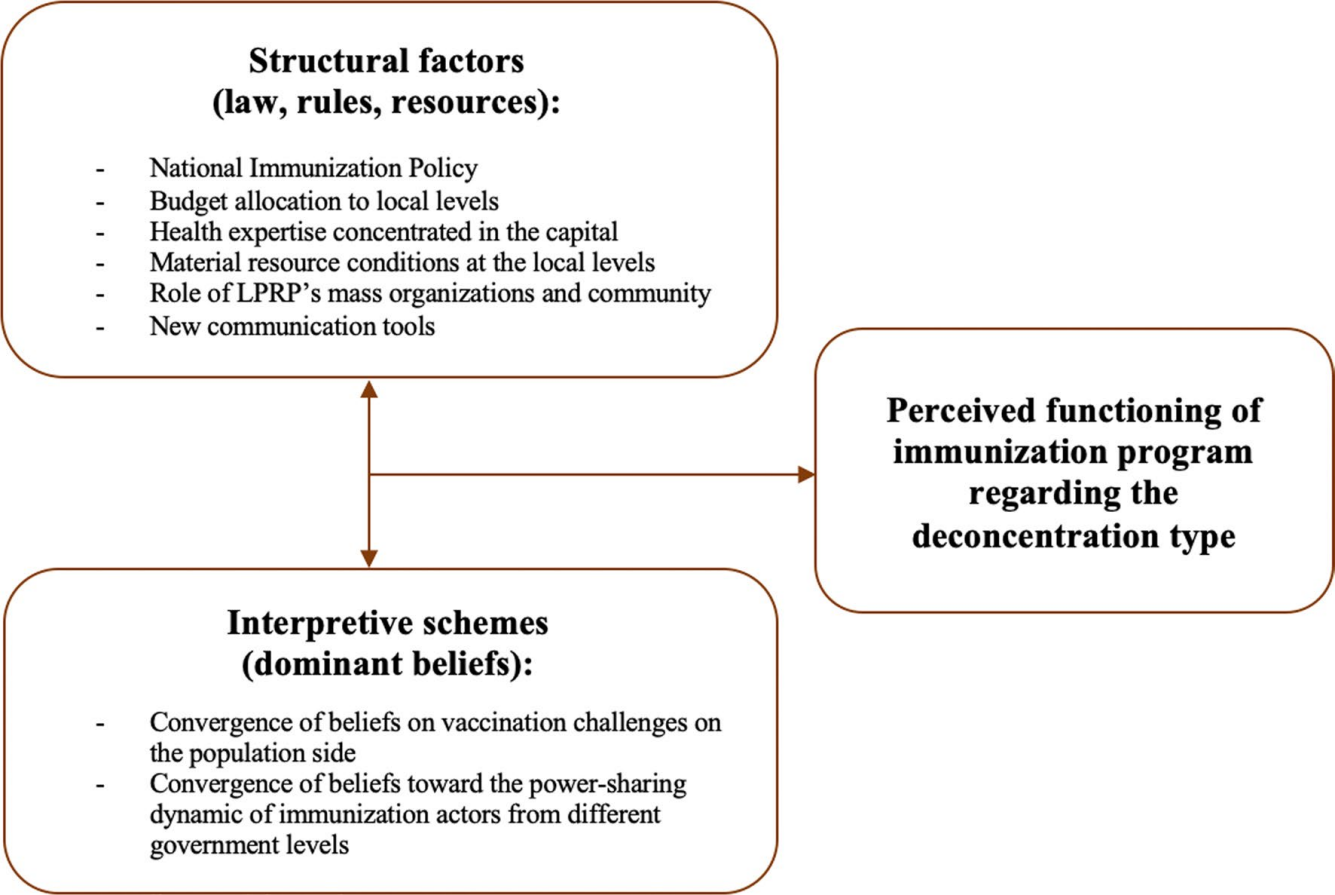
Social and organizational determinants associated with the decentralization type of immunization program in Lao PDR

Several factors related to either structure or interpretive schemas emerged from the data analysis (Fig. 1).

### Institutional structure of the immunization program in Lao PDR

Six main institutionalized factors associated with the structure of the immunization program, emerged from interviews and documents: (1) the national immunization policy; (2) the budget allocation and role of the GAVI international alliance for vaccines; (3) the human resources and expertise; (4) the material resources at the local levels; (5) the role of the LPRP’s mass organizations and community; and (6) the new communication tools.

#### The national immunization policy

The National Immunization Program (NIP) was launched as the Expanded Program on Immunization (EPI) in 1979 as a pilot-project in two provinces and gradually extended to cover all provinces in 1989 [30]. Administered mostly in the first year of life, routine vaccination is provided free of charge to children under one year old [29].

The program’s legitimacy is based on a government decree issued by the Prime Minister in 1993, followed by the endorsement of the National Immunization Law by the National Assembly in 2018. The law led the government to produce several documents to frame the roles and responsibilities of agencies at different government levels, even encouraging lower-level governments to mobilize resources to achieve immunization targets [29, 53].

#### Budget

Lao PDR being a lower-middle income country, routine immunization heavily relies on external donors, primarily the GAVI Alliance, for funding vaccines, injection equipment, cold chain supplies, safety boxes, and other essential resources. These donors also assist in developing guidelines and planning for various aspects of the immunization program, ensuring adherence to established norms and scientific standards [29, 30, 53]. Their contributions establish a formal structure for the program.

However, challenges arise in budget sourcing and distribution, impacting the program's functionality despite its well-thought-out organization. Immunization funds must be approved and distributed through the central level, resulting in delayed budget transfers from the MoH, especially in remote regions [28, 53]. Majority of local-level participants who raised this point emphasize that while they can request more funds from their local authority, ultimately all decisions are made at the central level.

The system is also fragile. During the COVID-19 pandemic for example, nearly all participants emphasize that routine immunization activities encountered disruptions, including delayed delivery schedules and limited outreach services at the community level. The focus shifted to vaccinating the entire population against COVID-19, leading to an extension of support from the NIP and donors like GAVI (pushing the withdrawal timeline from 2022 to 2023).

Yet, the biggest preoccupation raised by nearly all participants, is the fact that the vaccination program funding sources will have to change soon. Lao PDR aspires to reach the upper-middle income status by 2026. If this objective is reached, the government will not be able to benefit from the GAVI’s programs for lower-middle income countries, as it does presently. It will have to plan self-financing or securing external loans [25]. There is a concern widely shared among all participants regarding the capacity to guarantee enough funding once GAVI withdraws most of its support.

#### Human resources

Lao PDR grapples with a persistent challenge due to a shortage of vaccination staff at peripheral levels. The majority of participants at district and health center levels reveal that limited government staff at their facilities hinder effective operations. These remarks are supported by official documents [52, 53]. This is particularly evident when health workers are tasked with both vaccine administration and data management, along with other responsibilities such as Mother and Child Health (MCH) or curative services. Indeed, health workers are burdened with numerous responsibilities that extend beyond vaccine-related activities, as stated in the following quotation:

*“Nowadays, there are more update knowledge for newly graduated, it would be good if they come to work with us, it would reduce our works. However, the problem is the quota number, it depends on the upper level (central government) for the divided number, it is a problem. In our section, there are a lot of micro plans, so each of staff have to work for so many things” said by participant from provincial level *(*P7*)

Added to this dilution of expertise, according to the majority of participants and official documents, are other challenges, notably with regard to data management technology adoption by some health workers, in particular the older ones and English proficiency. Indeed, these reported issues have been shown to negatively impact the quality of immunization data management and reporting systems [29, 52]. Some participants at district and health center levels mention that immunization data are still recorded manually in notebooks or on blue boards mounted on walls in some districts and most health centers. This is shown in the following quotation*:*

“*When we report the daily vaccination rates, we write it, then take a picture of it and then send it. Then, the district would enter the data for us. Mostly they (DHOs) said that they (central level) would like us to enter the data ourselves, but mostly the data is wrong when we enter it ourselves, sometimes there is a duplicate data for just one person. The data is repeated*”* said by participant from health center (P19).*

Finally, the majority of participants at the local level acknowledge the shortage of human resources within vaccination teams, which often leads to communication challenges between vaccinators, who typically only speak the Lao language, and communities that predominantly speak other languages. While these teams frequently utilize interpreters, these interpreters often lack the specialized knowledge needed to resolve ambiguities effectively. As a result, interpretations tend to be largely literal and may not fully address underlying complexities.

#### Material resources

Immunization programs heavily depend on stable material resources, including cold chain equipment and reliable transportation for vaccines to reach all administrative levels. In Lao PDR, ensuring the availability of these resources for the immunization program is challenging due to factors like poor road conditions and budget constraints, particularly affecting the delivery of materials to remote district and health facility levels [30, 52]. Indeed, some participants at the local levels report difficulties in providing vaccine due to shortage of generators and vehicles. Nearly all participants agree that local authorities have insufficient means to adequately supplement immunization teams with materials provided by the central level, particularly impacting the maintenance of the vaccine cold chain.

#### LPRP’s mass organizations

The mass organizations are the arm of the LPRP in the community. They are involved in many health activities, including those funded and managed by external organizations.

In communities with distinct languages and traditions, mass organizations facilitate the smooth functioning of immunization services by leveraging their community knowledge [29, 53]. The LPRP, through its mass organizations and its representatives in health organizations, has the ability to closely monitor the activities carried out in the health system. Nearly all participants from the district and health center levels highlight that in "*difficult villages*", where villagers either do not access healthcare facilities for vaccines or disappear during outreach services, health center staff need to report these issues to LPRP representatives at the district health office. If solutions are ineffective, problems are referred to the provincial level, as stated in the following quotation:

*“At the village level, this includes the head of village and other heads of village Unions (mass organizations of the* LPRP*). At the district level, there are the committee for health including in the three unions, the Youth’s, the Women’s, and Lao Front’s Unions. In case we face some difficulties, we couldn’t get the cooperation from villagers as our target population, so we need to contact the district and village authorities together with all the Unions to come helping us” said by participant from provincial level (P7).*

#### Communication tools

Widespread access to smartphones and affordable internet services, platforms like Facebook and WhatsApp have changed the way communication take place in the Lao healthcare system. WhatsApp, in particular, is identified by almost all participants as a game-changer, enabling horizontal and vertical communication among healthcare workers. Nearly all participants emphasized how technology, especially social media, has transformed the once rigid hierarchical structure of interlevel communication in the Lao healthcare system. This technology has empowered staff at all administrative levels to engage in informal and less hierarchical exchanges. This can be shown in the following quotation:

“*In our office, we have WhatsApp groups. The first group includes the head of units, and the other group is for reports. In order to coordinate with provincial level, there is also an EPI group. It is the province who created this group and who included all the district EPI staff. For me, I am also in the group of the district party committee, and of the district council. When there is anything from provincial council committee, they would send it via this group*”* said by participant from district level (P14).*

### Interpretive schemes influencing the functioning of immunization program in Lao PDR

Interviews with the participants reveal a striking convergence between the interpretive schemes. Regardless of the governance level, respondents collectively comprehend the objectives and challenges of the immunization program and its functioning. This alignment manifests in two key elements derived from the data analysis: (1) beliefs on vaccination challenges; and (2) beliefs on power-sharing dynamic in Lao PDR.

#### Beliefs on vaccination challenges

In the realm of immunization, the acceptance of vaccination stands as a cornerstone of immunization activities. In culturally diverse countries like Lao PDR, communities vary widely in cultures, traditions, practices, and languages. Health workers at all levels of governance share a prevailing consensus. All participants from district and health center levels emphasize that in so-called risky districts, factors such as ethnicity, poverty, and maternal education level influence child vaccination rates. These influences stem from families' perceptions regarding the benefits and potential side effects of vaccination. This observation is also supported by the majority of participants at central and provincial levels.

#### Beliefs on power-sharing dynamic

More than half of the participants recognize the importance to take into account the fact that the expertise in immunization is concentrated in the capital and that provinces are generally devoid of expert resources. It is this aspect which explains above all why the sharing of power with regard to the immunization program is not conflictual. Yet, these participants also emphasize that beyond formal hierarchies and established customs, there is room for direct and indirect discussion across different levels of authority. They all describe how in Lao PDR, a country where the low population density means that the probability of knowing each other when working in the health system, whatever your position, is great. Moreover, a country like Lao PDR where social events that bring the community together are frequent, there are a considerable space for interindividual interactions unconstrained by hierarchical considerations. This coexistence between respect for a hierarchy traditionally based on age, social position and level of education, and a family attitude in relationships between people who know each other, whatever their position in the social hierarchy, is a “*Lao way*” which gives a unique color to the dynamic of power sharing in the Lao health system.

Furthermore, the majority of participants at the local level recognize the positive impact of socioeconomic changes, which result in particular in a gradual increase in the number of professionals with knowledge and skills who establish themselves in the provinces. These participants express increased confidence in a system which is increasingly able to provide quality training to future health professionals.

Finally, one notes that all participants emphasize that the COVID-19 pandemic has ushered in a new chapter in how individuals across different government levels engage, impacting the power-sharing dynamic within the Lao healthcare system. The use, in parallel with that of WhatsApp, of online platforms like Zoom and Google Meet, has increased online means to carry out exchanges between professionals located at various levels of governance. According to nearly all participants, this shift to online communication has transformed the traditional concept of meetings in Lao PDR. Previously confined to solely face-to-face interactions often followed by social activities, meetings now can be conducted virtually. New norms and practices have been put in place.

In summary, these socioeconomic changes have influenced the beliefs regarding the power-sharing dynamic among immunization actors across all government levels.

## Discussion

The neo-institutionalized theory allowed to identify institutionalized factors that are related to the effect of decentralization on the functioning of the immunization programs in Lao PDR. Among those, three stand out as having a foreseeable impact on the expected evolution of a system that has shown remarkable stability since the advent of Lao PDR: (1) the sociopolitical context that delineates the distribution of power among different levels of governance; (2) the resource availability of a lower-middle-income country; and (3) the use of new technologies by healthcare professionals.

### Lao PDR sociopolitical background

Lao PDR is a one-party state led by the Lao People's Revolutionary Party (LPRP). This party adheres to the Marxist-Leninist ideology, which emphasizes equality among various population groups, with particular attention to marginalized communities [55]. The ideology also promotes the concept of *democratic centralism*. The democratic component of the concept allows for open discussion and participation within the party. The centralism component ensures unity and discipline in decision-making process, providing the party’s leadership the responsibility for the final decision-making process [7, 55]. In Lao PDR, the central level, in accordance with this political conception of the state, detains higher control and power [8]. This is further compounded by the distinctive features of the Lao context, where the country's cultural traditions in addition to the political one, is marked by a strong respect for social hierarchy based several factors that include education level, and position in the bureaucratic hierarchy of the government apparatus [7, 55, 56]. In addition, the sociopolitical dynamics of Lao PDR are influenced by the Confucianist ideology, prevalent in Pacific and East Asian countries, including Lao PDR [57–59]. This reality is compounded by the Confucian custom of upholding hierarchical systems that prioritize the directives of individuals perceived to hold higher social and political statuses, thereby promoting political centralization.

This sociopolitical context strongly shapes power distribution in immunization efforts across government levels in Lao PDR. Within the Lao healthcare system, decision-makers at each tier are LPRP members, highlighting the political sway over public health matters. However, the voices of members of mass organizations from villages play a crucial role in decision-making within the LPRP, from local to central levels giving space for local concern about how to adapt programs to the realities of the communities. Similar to other single-party socialist nations like Vietnam and Cuba, local authority unions serve as grassroots agents overseeing health activities within their regions, significantly shaping health intervention outcomes at the community level [60, 61]. In Lao PDR, it is worth emphasizing that few public health activities are carried out without the consensus and support of mass organization members operating at the community level. These members come from the communities and therefore expected to have the capacity to understand and express local preoccupations. Their involvement gives a voice to the community, but also strengthens the dynamic of power lead by the central level.

In brief, the country's sociopolitical backdrop ensures consistency between the structure underpinning the activities deployed in the public health system, and the interpretative schemes shared within it. This suggests a continuation of stability [62].

### Resource availability

Lao PDR's immunization program, largely supported by external donors like GAVI, will soon faces a new budget situation caused by the country's economic transition to the upper-middle-income status [63, 64]. Indeed, Lao PDR is currently in the GAVI phase known as "accelerated transition" indicating that the country has surpassed GAVI’s eligibility criteria. The national government is now expected to contribute with at least 35% of the vaccine costs [65, 66]. The capacity of provinces to raise funds for immunization programs is currently quite unlikely. Lao PDR does not have the economy strength to allow local governments more autonomy in financial matters, as seen in China for example [67, 68]. The future seems to lead the country to perpetuate the deconcentration type of its healthcare decentralization. This prospect of maintaining the decentralized state that currently supports the immunization program is also reinforced by the fact that the country lacks specialized professionals, and skilled vaccine administrators and data managers at the district and health center levels [29]. Technical schools and universities are also in a relatively short period of time unable to train enough professionals to offset the significant shortage of human resources. Coupled with the current state of the country's financial resources, there is limited capacity to increase the number of government staff to meet nationwide needs, particularly in peripheral areas. In short, all respondents argued that the state of resources available in the country reduced the options for empowering local authorities to manage public health programs. There is therefore consistency between structure and interpretative schemes that are shared within this system. The resource situation points to continued stability.

### Use of new technologies by health professionals

In a country like the Lao PDR where the line between personal and work life is, for cultural and traditional reasons, often blurred in contrast to Western countries [69], many healthcare personnel frequently connect on social media platforms like Facebook and WhatsApp, sharing both professional and personal content including personal texts, photos and videos. With 3.60 million social media users in the country, constituting 49% of the population, these platforms play a significant role in communication. Helped by the COVID pandemic, WhatsApp has become the primary tool for team communication, used by 87% of healthcare personnel [70]. Additionally, WhatsApp has become a widely relied-upon tool for both formal and informal communication among healthcare personnel across the country. This new dynamic is also supported by the fact that healthcare personnel in the regions are increasingly well educated and comfortable using the new possibilities offered by information technology to understand the health problems of populations and seek appropriate solutions. This vertical and horizontal connectivity within WhatsApp groups at different authority levels streamlines coordination and fosters collaboration among personnel. Furthermore, this lateral interaction within these groups introduces a new dynamic that changes the traditional hierarchical norms of a decentralized system in a single-party state. This shift toward technology and decentralized platforms represents a significant transformation in the landscape of the healthcare system in Lao PDR.

The use of communication technology is expected to prompt professionals to reconsider their interpretative schemes, regarding the sharing of responsibilities between government levels. While the structure supporting decentralization has seen minimal change over the last 50 years, is not expected to drastically change, new interpretive schemes might replace the current ones, thanks to the advancements in technology and the development of human resource. At the local level, gradual decreases in human resource shortages, an uptick in graduates returning to their home provinces, and an increased use of modern communication technologies are anticipated. These shifts are likely to alter beliefs regarding the capacity of local resources to assume greater responsibilities. Though these changes may not fully resolve resource deficits, they may empower well-trained individuals at the local level to exercise more autonomy in managing immunization programs adapted to local populations. This transition toward greater local responsibility, informed by local insights and experiences, holds promise for enhancing immunization coverage within communities.

## Limitations

This study has some limitations. First, as with any qualitative study, unconscious subjectivity and bias among researchers might have influenced some interpretations. However, the risk is mitigated by ensuring four elements of results validity, particularly triangulation of information from interviews and document reviews, as well as independent analyses conducted by two researchers.

A second limitation is the generalizability of the study results. They may not apply to all public health programs in Lao PDR given that the diversity and regional variations within the country may lead to differences in the implementation and functioning of the immunization program across provinces. These results may also not apply to other single-party socialist countries which, although similar in political terms to Lao PDR, sometimes differ considerably in terms of ideological orientation, socioeconomic background, international relations, foreign policy, and degree of political decentralization/centralization.

## Conclusion

Unprecedented access to quality higher education and the increasing use of social networks might be altering power dynamics between local and central governments in the country, through their effect on the coherence between a structure that supports the deconcentrated form of decentralization and that has changed little over the last 50 years, and up-to-now widely shared interpretative schemes inside the healthcare system. This expected rebalancing of power relations might reshape the distribution of responsibilities devolved to different levels of government for public health programs such as the National Immunization Program.

### Supplementary Information

**Additional file 1. **Interview guide.

## Data Availability

The datasets during and/or analyzed during the current study are available from the corresponding author upon reasonable request.

## Abbreviations

CSOs: Civil society organizations
DHOs: District health offices
DTP3: Diphtheria, tetanus, pertussis
EPI: Expanded program on immunization
GAVI: Global Alliance for Vaccine and Immunization
Lao PDR: Lao People’s Democratic Republic
Lao TPHI: Lao Tropical and Public Health Institute
LPRP: Lao People’s Revolutionary Party
MCHC: Maternal and Child Health Center
MCH: Mother and child health
MoH: Ministry of Health
NGOs: Nongovernmental organizations
NITAGS: National Immunization Technical Advisory Groups
NIT: Neo-institutional Theory
PHDs: Provincial Health Departments
