# Supplementary material for: Decentralization and immunization program in a single-party state: the case of the Lao People’s Democratic Republic
Source: Trop Med Health. 2024 May 7;52:35. doi: 10.1186/s41182-024-00601-8 (PMC11075326; doi:10.1186/s41182-024-00601-8)
Supplement: Supplementary file 1 — Additional file 1. Interview guide. [file 41182_2024_601_MOESM1_ESM.docx]

**Interview Guide**

**Introduction**

Can you introduce yourself by telling us what organization you work for, what your role is and what your responsibilities are within that organization? Then, can you tell us how your activities are related to immunization?

**General question**

This meeting will focus on the division of responsibilities between the different levels of government and their impact on the National Immunization Program (NIP).

To begin, we would like to hear from you on this topic. Please tell us anything you would like me to know about the division of responsibilities between governments and the immunization program in Lao PDR.

**Specific questions**

- Who are the national and international stakeholders involved in the NIP?
- How do you perceive the effectiveness of the NIP in the country and why?
- How does the division of power and responsibilities between different levels of government influence the functionality of the NIP in Lao PDR?
- How do laws and administrative regulations influence the impact of power and responsibilities sharing between central authorities and provincial government on the functionality of the NIP?
- How do available human and material resources influence the impact of power and responsibilities sharing between different levels of government on the functionality of the NIP?
- What are the values, ideas, beliefs that, beyond laws and structures, influence the impact of power and responsibilities sharing between different levels of government on the functionality of the NIP?
- According to you, how do the changes that Lao PDR is experiencing with economic development influence the impact of power and responsibilities sharing between different levels of government and what are the consequences on the functionality and the effectiveness of the NIP?

**Before ending the interview**

Ask if the person has anything to add that he/she may have forgotten to mention. Ask if the person has any documents from their organization regarding the NIP that would be interesting to consult. Ask if the person can identify an organization or individual who might be able to provide information that differs from or complements the information provided.

**Acknowledgements.**
